# The Effect of Microwave Radiation on the Solidification of C-S-H Gels: Its Influence on the Solidified Cement Mixtures

**DOI:** 10.3390/gels9110889

**Published:** 2023-11-10

**Authors:** David Průša, Stanislav Šťastník, Karel Šuhajda, Kateřina Svobodová, Tomáš Žajdlík, Klára Hobzová, Miloslav Novotný

**Affiliations:** Institute of Building Structures, Faculty of Civil Engineering, Brno University of Technology, Veveří 331/95, 602 00 Brno, Czech Republic; stastnik.s@fce.vutbr.cz (S.Š.); suhajda.k@fce.vutbr.cz (K.Š.); 234215@vutbr.cz (K.S.); zajdlik.t@vutbr.cz (T.Ž.); 157465@vutbr.cz (K.H.); novotny.m@fce.vutbr.cz (M.N.)

**Keywords:** C-S-H, gel, microwave exposure, radiation, material drying, mechanical properties, compressive strength

## Abstract

The present paper deals with the properties of hardened cement mixtures that have been exposed to microwave radiation. Microwaves fall under electromagnetic waves (EMW), and the main reason for using EMW radiation is to accelerate the drying of concrete as well as to reduce the time required to obtain the handling strength after it is removed from the mould. This paper is divided into two main parts. In the first part, three sets of cement samples were made. One set of samples solidified naturally in air and the second and third sets of samples were exposed to EMW radiation, with different exposure times for each. The solidification was then stopped, and the representation of the major minerals was experimentally determined. The second part of the experiment focuses on the properties of the hardened cement mixtures, both in terms of strength and physical properties. The experiment was carried out on two sets of samples. Each mixture was exposed to EMW radiation, the main differences being the exposure time and the position of the samples relative to the EMW generator. The aim of the experiments is to determine the resulting mechanical properties of the samples in comparison with those that were subjected to normal solidification in air. The data from these experiments suggest that microwave radiation can be used to accelerate the curing of concrete specimens, obtaining the handling strength in a relatively short time, but a reduction in the resulting strength can be expected compared to the reference specimens.

## 1. Introduction

Concrete is the most widely used building material in the world, with production increasing every year [1]. There are basically two main methods of working with concrete. The first is in situ, the method of pouring concrete on site, and the second is the use of precast concrete units, i.e., they are produced in a factory, transported to the site, and then used to build complete structures [2,3,4].

Currently, there is a growing demand for precast concrete units [5]. They are produced in a controlled environment, which ensures a higher accuracy of proportioning the material and consequently the overall quality. On-site assembly can be very fast, which also ensures an altogether faster completion of the construction process. At the same time, dust and noise during construction can be limited [6]. The disadvantages of this method include the need to increase the initial strength of the precast concrete, which is achieved by thermal curing, where it is necessary to provide the energy needed to accelerate the curing [7]. It is therefore desirable to develop a highly efficient and environmentally friendly accelerator that does not compromise the long-term properties of the concrete.

Microwaves are part of electromagnetic waves (EMW), and one of the possible usages of microwave radiation in construction practice may be in the curing of concrete, specifically, accelerating the setting of concrete in its initial stages and gaining handling strength. This would provide the opportunity to remove the concrete blocks from the moulds faster and ensure a higher production rate. Pilot studies were already carried out in 2002. They were undertaken by the Commonwealth Scientific and Industrial Research Organisation (CSIRO), an Australian company led by Dr Swee Liang Mak who dubbed the research Low Energy Accelerated Processing (LEAP). The result of this pilot research was that microwave radiation can be used to accelerate the setting of concrete, and the difference in strengths between reference concrete samples and concrete samples where accelerated setting had taken place was within 10% after 28 days, and after 4 h and 30 min, a strength of about four times that of the reference samples was even achieved at a water factor of 0.5 [8,9].

The presented research builds on the initial, previous research and extends it further, in particular by analyzing the effect of microwave radiation on C-S-H gels during concrete setting and subsequently on the properties of the hardened concrete. The distribution of individual minerals is investigated by X-ray analysis and their structure is studied by microscopy. Furthermore, the results of the previous experiments are methodically verified by checking the strength and absorption characteristics.

### 1.1. Literature Review

The main purpose of this work is to verify the possibility of using microwave radiation in the hardening of concrete. The reason for this experiment is the desire to speed up the hardening process in order to reduce the amount of time the products have to be stored in moulds before they gain handling strength. By speeding up the curing and obtaining the handling strength earlier, this would ensure higher concrete production.

**Figure 1:**
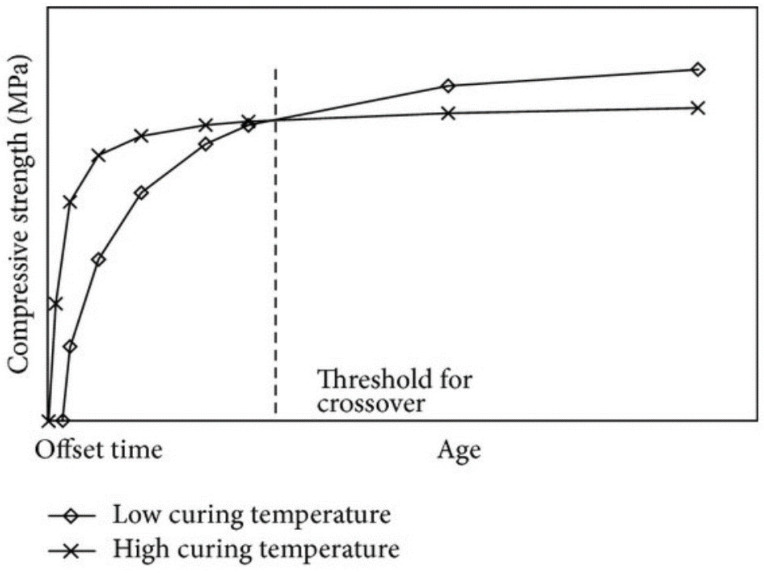
Strength development of concrete due to different initial curing temperatures [11].

Efforts have been made in the past to speed up the curing of concrete and there are a number of ways to speed up the process. However, the resulting concrete properties vary considerably from reference values, with standard concrete strengths being 20% lower where the microwave acceleration of curing has taken place, which can be seen in Figure 1 [10].

Researchers [12,13] have described that increasing the curing temperature of concrete increases the rate of strength gain early in the curing process but decreases long-term strength (see Figure 1). Garcia and Sharp [14] also report that concrete exposed to high curing temperatures exhibits accelerated hydration and thus an uneven distribution of hydration products. Such exposure produces less porosity at early ages and high porosity at later ages. This results in increased compressive strength at an early age and reduced compressive strength at a later age. Ogirigbo and Black [15] described that concrete cured at an elevated temperature of 38 °C had less porosity and greater compressive strength after seven days than concrete cured at 20 °C. However, after 28 days, the porosity of the concrete cured at the elevated temperature increased and the compressive strength decreased compared to the concrete cured at normal temperature. Ezziane et al. [16] pointed out that under an elevated temperature, cement mortars and concretes have a more porous and thus worse physical structure than those formed at normal room temperature. They also stated that high temperatures increase the strength gain at early ages, while at later ages, a substantial amount of the hydrates formed do not have enough time to arrange themselves properly.

Kjellsen and Detwiler [17] report that the 28-day compressive strength of samples cast and cured at a temperature below 5 °C is approximately 80% of that of samples cast and cured at 21 °C to 46 °C. The compressive strength at later ages would be lower at higher casting and curing temperatures. According to Newman and Choo [18], the compressive strength at later ages of heat-treated CBM (concrete or cement bound material) is generally lower than the compressive strength of conventionally cured samples. This is due to the lower reaction rate at lower temperatures, and it means that mortars and concretes should be cured for a longer period of time to obtain the required strength. Khan and Abbas [19] reported that a rapid reaction at elevated temperatures gives relatively high early strengths, but long-term strength and durability are commonly reduced.

Lothenbach et al. [20] state that the hydration of cement is strongly influenced by temperature. A higher temperature during hydration leads to high strength in the early stages. At later stages, the strength of cements hydrated at a high temperature decreases compared to cements hydrated at room temperature. Sajedi and Razak [21] reported that temperature changes caused by either heat of hydration or changes in the external environment significantly affect the mechanical properties, especially the compressive strength of concrete and mortar at an early age. Kim et al. [22] demonstrated with experimental results that concretes and mortars exposed to high curing temperatures obtain higher compressive strengths and split tensile strengths at early ages but lower compressive strengths and split tensile strengths at later ages compared to concretes exposed to room temperature curing.

Namarak et al. [23] indicate that increased curing temperature can accelerate both pozzolanic and hydration reactions and achieve higher compressive strength in the early stages of hydration. However, at later stages, mortars and concretes cured at a high temperature achieve compressive strengths lower than those at a normal curing temperature. Similar behavior was also observed by Ezziane et al. [16], with a reduction in compressive strength when mortars were subjected to high-temperature curing using natural pozzolan as a cement replacement [10]. These findings contradict the results of Dr. Swee Liang Mak, whose research, using microwave radiation to accelerate the setting of concrete, found that the compressive strengths of microwave-cured specimens were comparable to those of reference specimens [8].

Since cement is the binder component of concrete, this paper investigates and verifies the effect of microwave radiation on it, or more precisely, on C-S-H gel.

#### Microwave Energy

Microwaves refer to a portion of electromagnetic radiation with a frequency of 300 MHz to 300 GHz, corresponding to wavelengths from 1 mm to 1 m. More frequencies are allowed for industrial use, but in civil engineering the frequency of 2450 MHz with a wavelength of 12.2 cm is of particular interest. The microwave radiation causes heating, and the molecules become oriented according to their polarity in the electric field. When microwave radiation comes into contact with water molecules, electromagnetic energy is transformed, and the molecules are heated. Subsequently, other construction materials are heated [24]. The use of microwave equipment is quite safe, and a person can be harmed only by improper handling of the equipment or from direct exposure from a very close distance for at least a few minutes [25]. Strong microwave radiation poses a higher risk for human health, which is for this reason limited by several regulations, including one from EU (Directive 2004/40/EC) [26]. Some countries also have their own limits. Usually, the regulations specify the permissible electromagnetic field strength (from 7 V/m to 61 V/m) or watt density (from 0.1 W/m^2^ to 10 W/m^2^) [25].

**Figure 2:**
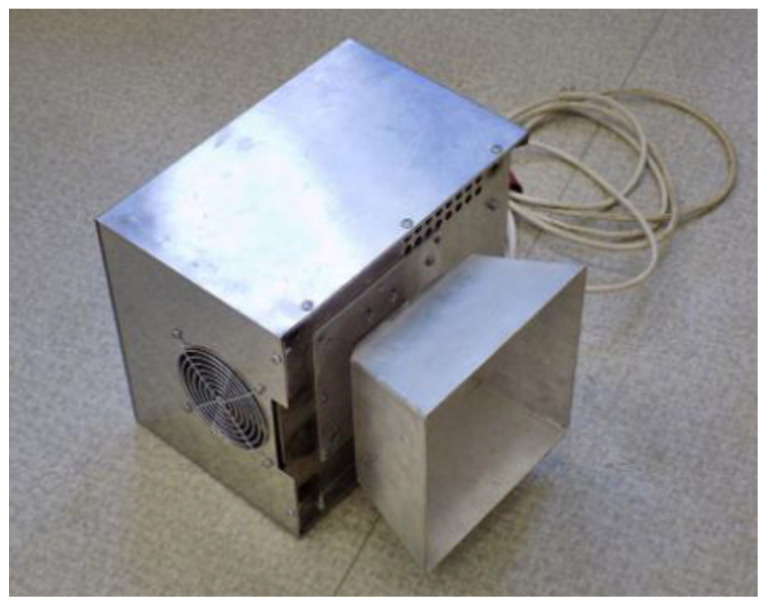
Examining power generator with horn antenna [25].

Almost every household has a microwave oven nowadays, and the concept of microwave heating is also used in many other sectors outside the food industry. For example, it is widely used in medicine, the aforementioned food industry, the military or aviation industry, as well as in the construction industry. Numerous studies have already been carried out in the field of construction, which have confirmed the possibility of using microwaves for drying construction materials [27,28,29,30,31]. The assembly used for irradiation consists of an energy source, an energy generator, a horn antenna, and a microwave intensity indicator, as shown in Figure 2 [24].

## 2. Results and Discussion

**Figure 3:**
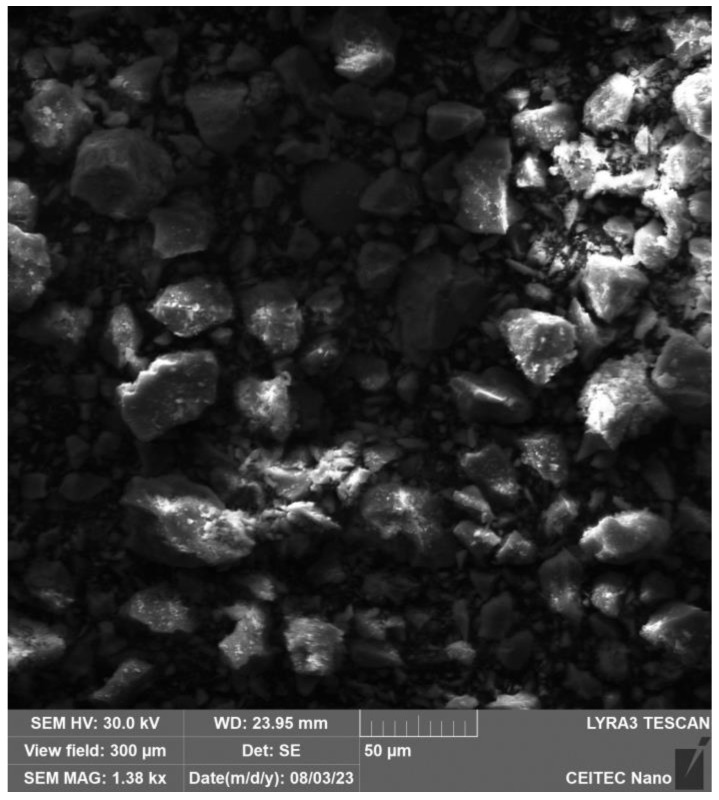
Electron microscope view of cement after 30 min of hydration in air.

**Figure 4:**
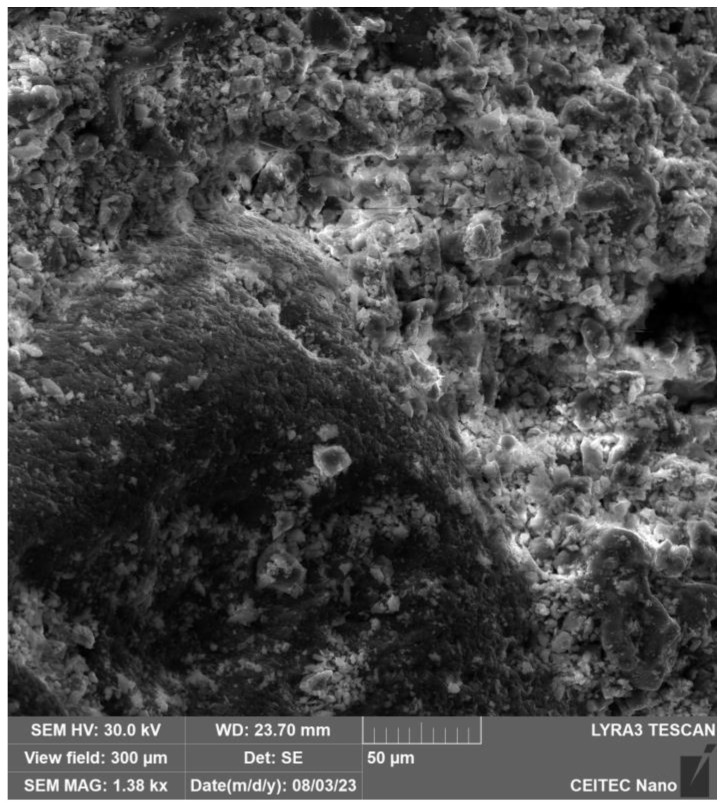
Electron microscope view of cement after 30 min of exposure to microwave radiation.

**Figure 5:**
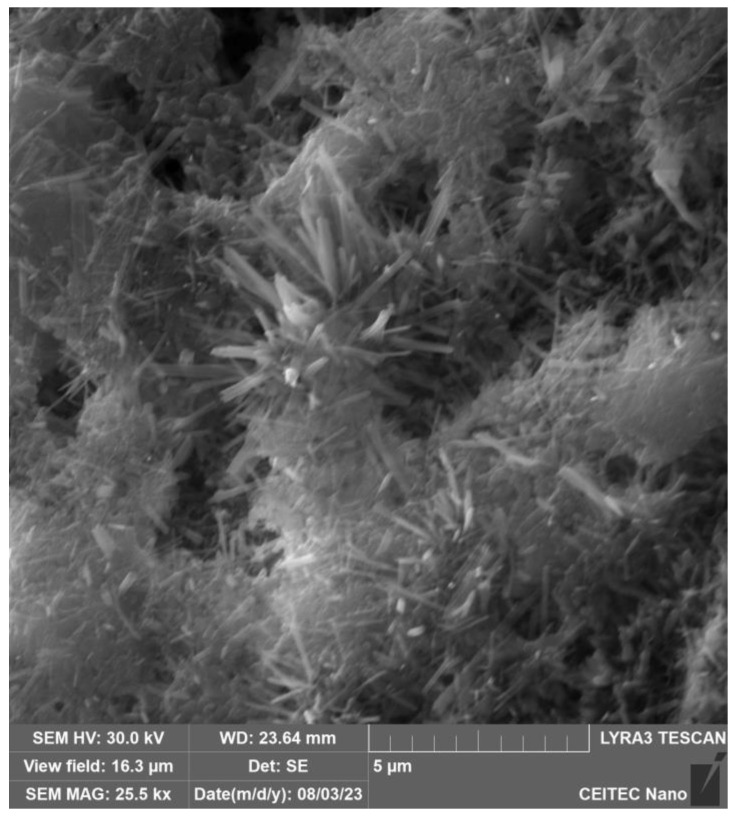
Electron microscope view of cement after 60 min of hydration in air.

**Figure 6:**
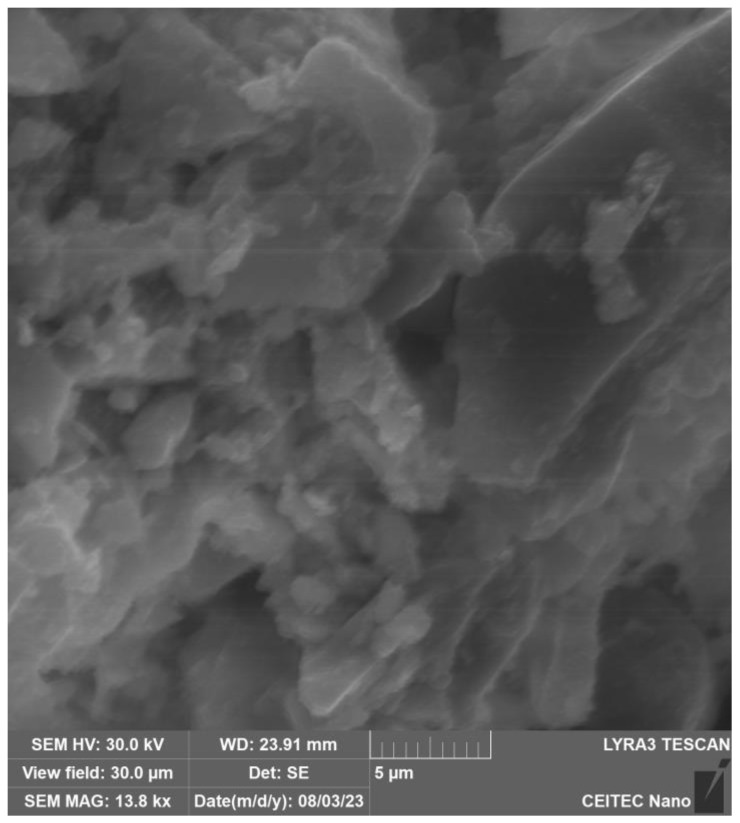
Electron microscope view of cement after 60 min of exposure to microwave radiation.

The resulting properties can be seen in Figure 3, Figure 4, Figure 5 and Figure 6. The designation of experiment 1 means that the concrete hardened and cured in a standard way in air and then in the water bath. The designation of experiment 2 means that the concrete was exposed to microwave radiation for 30 min and then immediately demoulded and, after setting to room temperature, was stored in the water bath. Experiment 3 indicates that the concrete was exposed to microwave radiation for 60 min and then immediately demoulded and stored in the water bath after settling to room temperature.

In Figure 3, the particle structure of the binder and filler, which have not yet dissolved into the solution, can be seen. Figure 4 and especially Figure 5 show the growth of crystalline neoplasms, where intense heating by microwave radiation allowed their formation from the saturated state of the solution. Figure 6 shows particles of the mixture that have not completely dissolved in the solution.

**Figure 7:**
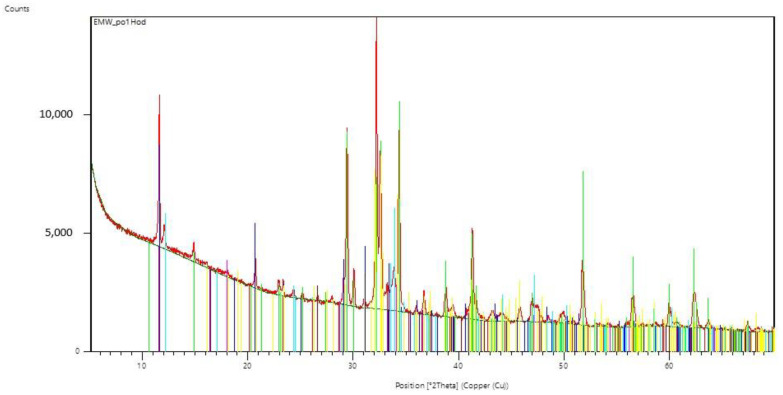
Radiograph of C-S-H gel samples that have been exposed to microwave radiation for 60 min.

**Figure 8:**
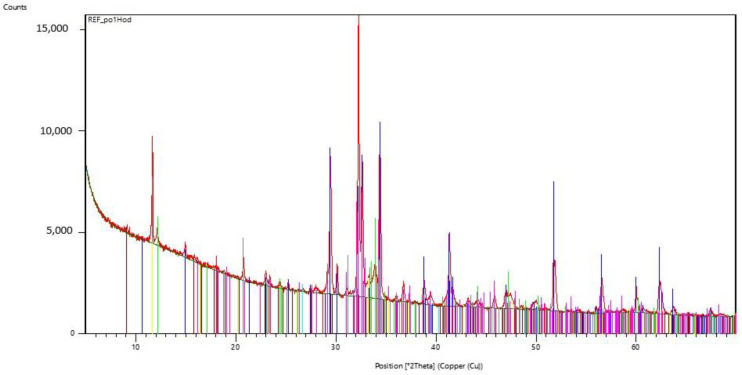
Radiograph of C-S-H gel samples not exposed to microwave radiation.

For two samples, the curing process was stopped after 60 min. One sample was exposed to microwave radiation and the other cured in the standard way. X-ray analysis was performed on these samples and the resulting radiographs can be seen in Figure 7 and Figure 8. Table 1 and Table 2 show the dominant materials found in the samples.

**Figure 9:**
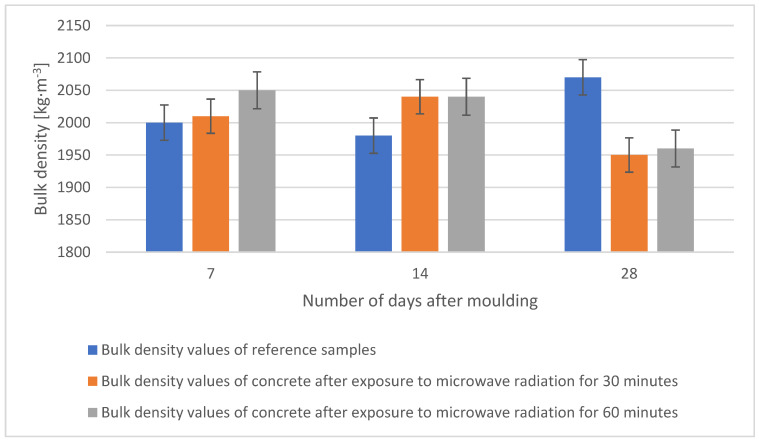
Comparison of the bulk density values of individual samples after 7, 14, and 28 days.

When comparing the fresh concrete bulk density values, the results do not show significant differences. The bulk density of hardened concrete was determined at the age of the samples of 7, 14, and 28 days. This test was carried out according to ČSN EN 12390-7 on 40 × 40 × 160 mm beams and it preceded the compression test. The test specimens were stored in a water bath prior to the test. The results and corresponding time histories of the bulk weights of all concretes are shown in the graph in Figure 9.

**Figure 10:**
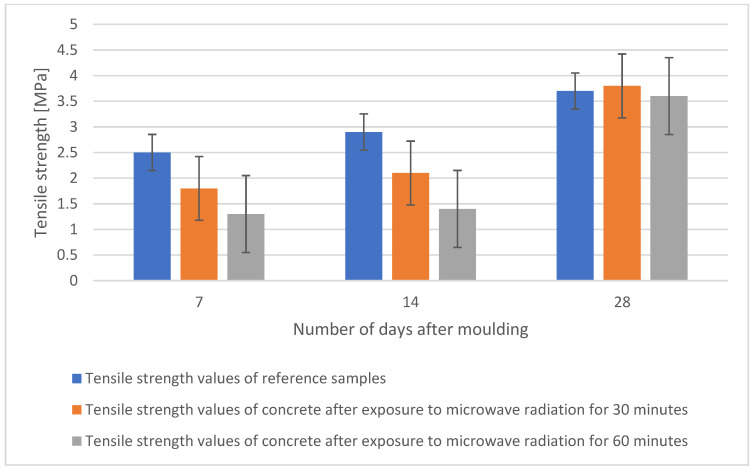
Comparison of tensile strengths of individual samples after 7, 14, and 28 days.

The tensile strength test was carried out according to ČSN EN 12390-5 on beams of 40 × 40 × 160 mm, and it preceded the compression test. The test specimens were stored in a water bath before the test and then dried in an oven at 105 °C. The tensile strengths of each specimen over time for all concretes are shown in the graph in Figure 10. The results show that after 7 days and 14 days the reference specimen has the highest tensile strength, but after 28 days the tensile strength results are comparable with a maximum difference of 3% between them.

**Figure 11:**
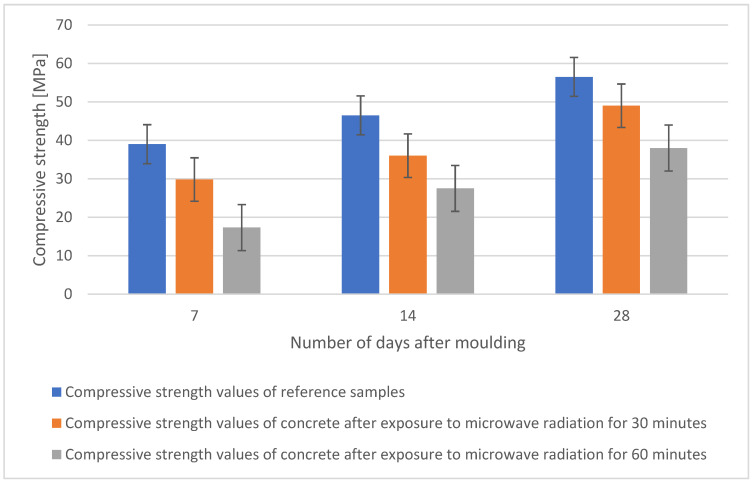
Comparison of compressive strength values of individual samples after 7, 14, and 28 days.

The compressive strength test was carried out according to ČSN EN 12390-5 on 40 × 40 × 160 mm beam fragments and was completed after the tensile strength test. The test specimens were stored in a water bath prior to the test and then dried in an oven at 105 °C. The compressive strength of each specimen over time for all concretes is shown in Figure 11. The results show that the reference specimen has the highest compressive strength after 7, 14, as well as after 28 days. The trend is maintained where the reference specimen always has the highest strength and any further prolonged exposure of the concrete to microwave radiation negatively affects the resulting compressive strength.

**Figure 12:**
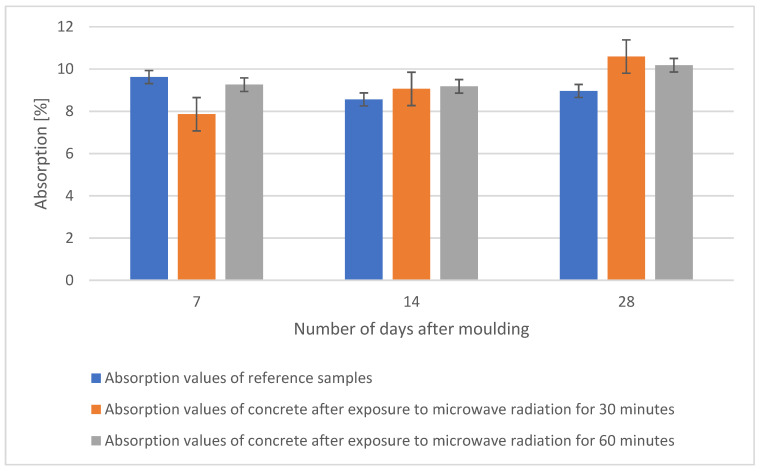
Comparison of absorption values of individual samples after 7, 14, and 28 days.

The absorption test was carried out according to ČSN EN 12390-5 on beams of 40 × 40 × 160 mm and preceded the tensile test. Prior to the test, the specimens were stored in a water bath and then dried in an oven at 105 °C, after which they were weighed and placed in a water bath for 24 h and weighed again. The values of water absorption of each sample over time are shown in Figure 12. The obtained values show that after 28 days the reference sample has the lowest water absorption value.

### Evaluation of the X-ray Analysis

It is evident from the radiographs taken that the used cements were silicate cements based on Portland clinker, of the system CaO-SiO_2_-Al_2_O_3_, Fe_2_O_3_, without significant representation of minor components. The analysis only captures the presence of crystalline phases, because glassy phases cannot be captured by this X-ray diffraction method.

**Figure 13:**
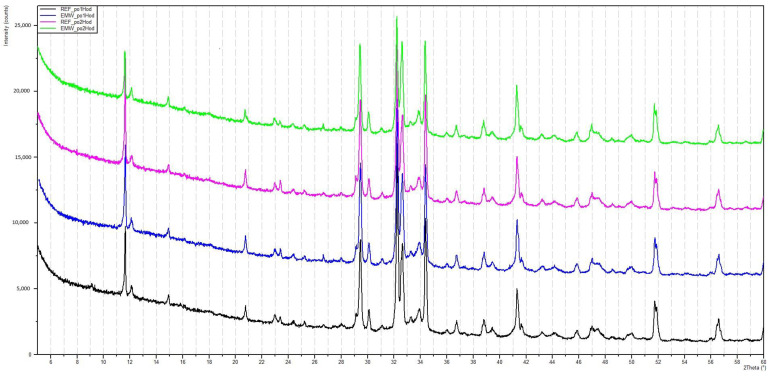
Display of individual experiments in a single radiograph.

In the initial phase, the hydration process is influenced by the EMW stress, which causes an increase in the temperature of the water present around the cement grains, namely the hydration rate applied by the reaction kinetics on the surface of the cement grains. Crystals of ettringite and calcium hydroxide are precipitated from the supersaturated solution. It is apparent from the course of the radiographs that no unexpected crystalline phases are formed. It seems that the effect of EMW radiation affects the reaction kinetics of hydration mainly in its initial phase. A comparison of the individual radiographs from the experiment can be seen in Figure 13.

The black curve represents the resulting radiograph of the reference sample after 30 min of hydration. The purple curve represents the resulting radiograph of the reference sample after 60 min of hydration. The blue curve represents the resulting radiograph of the sample that was exposed to microwave radiation for 30 min. The green curve represents the resulting radiograph of the sample that was exposed to microwave radiation for 60 min.

The rate of hydration, together with the amount of hydration heat released, depends on a number of factors, but the determining influence is primarily the mineralogical composition of the cement. The rate of hydration depends on the time of mixing of the cement with water; later, it decreases with time. It is known that the ratio of alite to belite can influence the increase in strengths and the development of the heat of hydration at the various stages of hydration. The age of the cement stone and the value of the specific surface area of the cement grains can be also considered as factors influencing hydration, since the depth of hydration is small relative to the largest size of the clinker grains in coarse-ground cements, for example.

## 3. Conclusions

Based on the conducted experiments and their subsequent evaluation, it was possible to confirm the effect of curing acceleration by microwave radiation on the mechanical properties of composite materials where cement is used as a binder. The data obtained show that microwave radiation can be used to accelerate the curing of concrete specimens, obtaining the handling strength in a relatively short time, but a reduction in the resulting strength can be expected compared to the reference specimens.

The experiment was conducted in three sets. In the first set, the concrete specimens that were cured in a standard manner in air were used. In the second set, the concrete samples were exposed to microwave radiation for 30 min. In the third set, the concrete samples were exposed to microwave radiation for 60 min. The resulting values of mechanical and physical properties of the samples can be observed in Table 3.

The resulting mechanical properties were determined by destructive strength tests. The tensile strengths of the specimens exposed to microwave radiation and the reference specimens were found to be comparable. Therefore, the main difference in the mechanical properties is mainly in the compressive strength. The experiments show that the resulting compressive strengths of the reference specimens are higher than those of the specimens exposed to microwave radiation, and at the same time a trend can be observed whereby the resulting compressive strength of the concrete decreases as the time of exposure to microwave radiation increases. The results suggest that the cause of this difference can be found in the higher temperature to which the concrete was exposed. The values of bulk density and absorption can be considered comparable for all samples and, in general, the resulting effect of microwave exposure on these properties of the samples cannot be clearly specified.

X-ray analysis indicated that the samples contain the same minerals in approximately equal proportions. In general, it cannot be said that microwave exposure has an effect on the representation of individual clinker minerals during hydration of the C-S-H gel. The display of the specimens can be seen in Figure 3 and Figure 4 and Section 4.2, where the gradual hardening of the concrete can be observed. It can be noticed that the concrete exposed to microwave radiation has a lower moisture content and the cement has hydrated faster.

The advantage of microwave heating in general is that the heating of the moisture contained in the porous system of the building material occurs directly in the material; this is accomplished through the absorption of microwave radiation by the moisture. With microwave heating, handling strength is achieved in a relatively short time and the process of producing concrete parts can be accelerated. Other advantages include that the microwave heating directly in the construction material is fast; that microwave drying can be applied to a wide range of building materials; and that microwave emitters are mobile and easy to operate.

Disadvantages include, for example, that the cost of purchasing a microwave generator is higher; that microwave radiation can be harmful to health and it is therefore not advisable to be near the irradiated material; and that it is not possible to continuously measure the temperature in the irradiated material because the temperature sensors are metal based, so they interact with the microwave radiation.

The experiments carried out can be considered as initial experiments and the paper is intended to serve as a stimulus for further research to address the current issue of prefabricated parts production and acceleration of their curing, for example, by reducing the water required for cement hydration.

## 4. Methods and Materials

The experimental study was carried out on several concrete samples. For the sake of transparency and reproducibility of the individual experiments, the mixture that formed the final concrete was always the same. Specifically, the concrete was a mixture where the binder was Portland cement CEM I 42.5 R, and the filler was glass sand and water. The representation of the different components in the mixture was in the ratio 1:3:0.5.

The main objective of the experiment was to investigate the effect of microwave radiation on fresh concrete during its setting and curing. A total of 48 samples of this mixture were made and placed in steel and silicone moulds with internal dimensions of 40 × 40 × 160 mm. A total of 24 samples were solidified and cured in a standard way in the steel moulds in air and 24 samples were exposed to microwave radiation.

**Figure 14:**
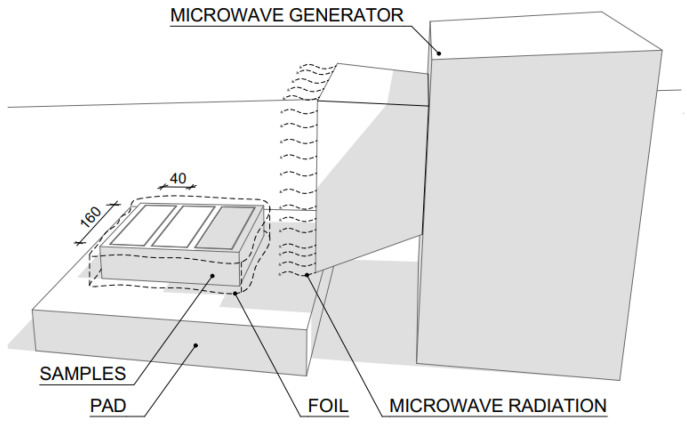
Scheme of the microwave generator assembly for concrete exposure.

Of these 24 samples, 12 were exposed to microwave radiation for a total of 30 min and the other 12 samples were exposed to microwave radiation for 60 min. The samples were placed 30 cm away from the microwave generator as shown in the following Figure 14.

These samples were further stored in a water bath for 7, 14, and 28 days and then subjected to strength tests, i.e., compressive and tensile strength, a water absorption test, and bulk density test.

In addition, 6 samples of fresh mixtures were made and placed in circular containers with a diameter of 80 mm and a depth of 10 mm. Three of these samples were solidified and hardened in a standard way in air, whereas the other three samples were exposed to microwave radiation. These samples were stopped from solidifying after 30 min using alcohol and subjected to X-ray analysis. The aim was to determine the representation of the different clinker minerals in the mixtures and to find differences in solidification by the two methods.

### 4.1. Equipment

Currently, several types of EMW radiation are used in building practice for drying damp buildings. Although a lot of systems dry masonry by simply connecting an EMW generator to a magnetron, the use of EMW antennas increases the efficiency of drying. Typical drying methods in construction practice use a rod- or funnel-shaped EMW antenna [32]. In the present experiment, only a funnel-shaped antenna was used. During drying, a unit equipped with a funnel-shaped antenna was attached to the masonry at distances of 0 to 50 mm and left in operation for a period of time at any chosen location. By gradually cycling the antenna, the entire area was dried. It is important to dry the individual locations for a shorter period of time and repeatedly, as otherwise rapid drying may result in the formation of subsurface pockets, with the result being that only the surface of the masonry is dried, and moisture remains in the depths of the material. Cyclic drying eliminates the formation of pockets and ensures the heating in the depths of the material. In this experiment, a Romill portable microwave generator, G1/2011, was used to irradiate the bricks. The voltage used was 230 V, 50 Hz, the power input was 1.5 kW, the microwave frequency was 2450 MHz, and the maximum microwave power was 1 kW [31].

### 4.2. Preparation of Samples

The individual components were portioned and mixed according to a fixed procedure. First the aggregate was dried, then the individual components were weighed to ensure that the concrete would have the same properties for all samples. The weighed components were dosed into the forced circulation mixer in the order of aggregate and then cement. This mixture was stirred for 90 s, which ensured a high homogeneity of the dry component. After the mixture was homogenized, the water was added. The fresh concrete was deposited into the moulds, which were wiped with a separating agent for easier demoulding. A total of two types of moulds with internal dimensions of 40 × 40 × 160 mm were used, namely a steel mould and a silicone mould. The compacting of the concrete was carried out by vibration—a vibrating shaker table was used for the three gang beam moulds. A total of 48 samples of this mixture were produced and placed in steel and silicone moulds with internal dimensions of 40 × 40 × 160 mm. A total of 24 samples were solidified and hardened in the standard way in the steel moulds in air and 24 samples were exposed to microwave radiation.

**Figure 15:**
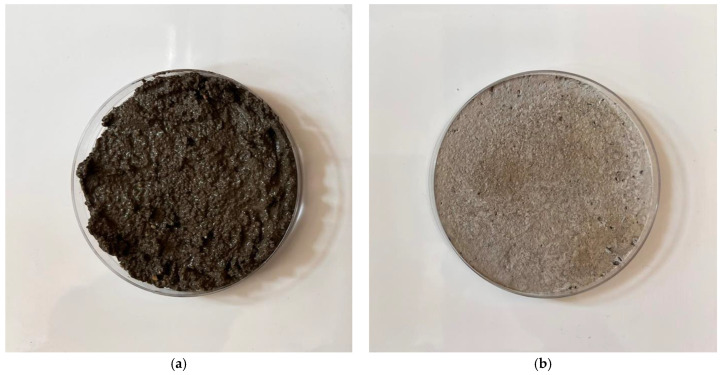
Photographs of samples after 30 min of curing. (**a**) Photograph of a concrete sample in a circular container that has cured in the standard way after 30 min of curing. (**b**) Photograph of a concrete sample in a circular container that has been exposed to microwave radiation for 30 min.

Of these 24 samples, 12 were exposed to microwave radiation for a total of 30 min while the fresh mixture was solidifying, and the other 12 samples were exposed to microwave radiation for 60 min. The samples were placed 30 cm from the microwave radiation generator as shown in Figure 15.

Demoulding of the specimens was always carried out within 24 h of placing the concrete in the moulds, and the specimens were legibly labeled and placed in the environments in which they were treated. One common treatment method was chosen for the concrete, placing the blocks in a water bath at 20 °C.

**Figure 16:**
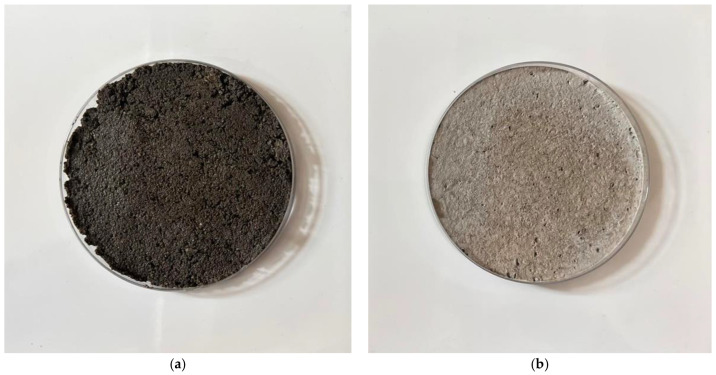
Photographs of samples after 60 min of curing. (**a**) Photograph of a concrete sample in a circular container that has cured in the standard way after 60 min of curing. (**b**) Photograph of a concrete sample in a circular container that has been exposed to microwave radiation for 60 min.

Figure 15a. shows a sample of concrete cured for 30 min using the standard method and Figure 15b. shows concrete cured using microwave radiation. Figure 16a. shows a concrete sample that has cured for 60 min in the standard way and Figure 16b. shows concrete that has been cured using microwave radiation.

### 4.3. Experimental Work Procedure

In order to allow the comparison of the properties of the different concretes, the same test methods and procedures were chosen for all samples. The overall set of experimental work can be divided into two parts.

The first part of the testing was aimed at determining the representation of clinker minerals in the concrete during setting. A total of 6 samples of fresh mixes were made and placed in circular containers with a diameter of 80 mm and a depth of 10 mm. Three of these samples were solidified and cured in a standard way in air and three samples were exposed to microwave radiation. These samples were stopped from solidifying after 30 min using alcohol and subjected to DTA analysis. The aim was to determine the representation of the different clinker minerals in the mixtures and to find differences in solidification between the two methods.

The second part of the test methods concerns hardened concrete, more precisely the determination of the physical and mechanical properties of hardened concrete. The compressive strength of the concretes was determined on beams of dimensions 40 × 40 × 160 mm on fractions of the solids after the flexural tensile strength test.

### 4.4. Placement in a Water Bath

The basic treatment method was a water bath. The test specimens were carefully and legibly labeled after demoulding and then placed in a container of potable water that was placed in a tempered laboratory. The water was kept at a constant temperature of 20 ± 2 °C. The test samples were kept in this bath until they were removed, superficially dried, and subjected to physical and mechanical tests.

### 4.5. X-ray Powder Diffractometry

For X-ray structural analysis, an X-ray diffractometer system Empyrean with an anode CuK alpha as a source of monochromatic X-ray radiation with Ni radiation filtration was used. Sample preparation consisted of interrupting the hydration process by dehydrating the samples after a definite time and agitating the samples in grinding mortar to the desired fineness of fragmentation.

### 4.6. Determination of Bulk Density (ČSN EN 12390-7)

The bulk density of the hardened concrete is determined by measuring the volume of the test specimen on the basis of its dimensions and then determining its mass. The preparation of the test specimens, the test procedure and the evaluation were carried out in accordance with the relevant standard ČSN EN 12390-7 [33].

### 4.7. Determination of the Flexural Tensile Strength (ČSN EN 12390-5)

The flexural tensile strength was determined on 40 × 40 × 160 mm beams placed symmetrically on the supports and uniformly loaded with a hydraulic press perpendicular to the compaction direction. The flexural strength value was calculated as the average of the three determined values. The test procedure and the evaluation were carried out in accordance with the relevant standard ČSN EN 12390-5 [34].

### 4.8. Determination of Compressive Strength (ČSN EN 12390-3)

The compressive strength of the concretes was tested on fractions of the test beams after the flexural tensile strength test. The loading was applied perpendicular to the compaction direction. The compressive strength value was calculated as the arithmetic mean of the values determined on the test beam fragments. For this test, the compressive strength was determined after 7, 14, and 28 days. The test procedure and the evaluation were carried out in accordance with the relevant standard ČSN EN 12390-3 [35].

### 4.9. Determination of Concrete Water Absorption (ČSN EN 206+A2)

The test blocks are weighed and then placed in a 105 °C oven for 72 h. After the required time has elapsed, the test blocks are retrieved and after cooling down to room temperature they are weighed again. The blocks are placed in a bath so that they do not touch. The bottom is covered with two wooden prisms serving as supports for each beam. The tub is then filled with water, 2 cm above the top surface of the blocks. At predetermined intervals over a period of 72 h, the beams are removed from the water, dried with a damp cloth, and weighed. After 72 h, the samples are placed in a pot of boiling water for 4 h and then weighed again. The weight increments are recorded in a log. The test procedure and the evaluation were carried out in accordance with the relevant standard ČSN EN 206+A2 [36].

## Figures and Tables

**Table 1:** The dominant minerals that were assessed by X-ray analysis in C-S-H gel samples that were exposed to microwave radiation for 60 min.

Display Color	Mineral Name	Chemical Formula
Blue	Gypsum	CaSO_4_·2H_2_O
Lime	Hatrurite	Ca_3_SiO_5_
Maroon	Quartz low	SiO_2_
Aqua	Brownmillerite	Ca_2_(Al,Fe)_2_O_5_
Fuchsia	Portlandite	Ca(OH)_2_
Yellow	Larnite	Ca_2_SiO_4_
Red	Tricalcium Dialuminate	3CaO·Al_2_O_3_

**Table 2:** The dominant minerals, which were assessed by X-ray analysis in C-S-H gel samples that were not exposed to microwave radiation.

Display Color	Mineral Name	Chemical Formula
Blue	Hatrurite	Ca_3_SiO_5_
Lime	Brownmillerite	Ca_2_(Al,Fe)_2_O_5_
Gray	Gypsum	CaSO_4_·2H_2_O
Maroon	Ettringite	Ca_6_Al_2_ (SO_4_)_3_(OH)_12_·26(H_2_O)
Aqua	Quartz low	SiO_2_
Fuchsia	Larnite	Ca_2_SiO_4_
Yellow	Tricalcium Dialuminate	3CaO·Al_2_O_3_
Red	Portlandite	Ca(OH)_2_

**Table 3:** The resulting values of bulk density, tensile strength, compressive strength, and water absorption of the samples after 7, 14, and 28 days.

Characteristic		7 Days	14 Days	28 Days	95% Confidence Interval Values
Volume weight [kg∙m^−3^]	Experiment 1	2000	1980	2070	±14.0
	Experiment 2	2010	2040	1950	
	Experiment 3	2050	2040	1960	
Tensile strength [MPa]	Experiment 1	2.5	2.9	3.7	±0.33
	Experiment 2	1.8	2.1	3.8	
	Experiment 3	1.3	1.4	3.6	
Compressive strength [MPa]	Experiment 1	39	46.5	56.5	±4.01
	Experiment 2	29.8	36	49	
	Experiment 3	17.3	27.5	38	
Absorption [%]	Experiment 1	9.62	8.56	8.96	±0.27
	Experiment 2	7.86	9.06	10.59	
	Experiment 3	9.26	9.18	10.18	

## Data Availability

All data and materials are available on request from the corresponding author. The data are not publicly available due to ongoing research using a part of the data.

## References

[1] Zou F., Zhang M., Hu C., Wang F., Hu S. (2021). Novel C-A-S-H/PCE nanocomposites: Design, characterization and the effect on cement hydration. J. Chem. Eng..

[2] Han Q., Wang D., Zhang Y., Tao W., Zhu Y. (2020). Experimental investigation and simplified stiffness degradation model of precast concrete shear wall with steel connectors. Eng. Struct..

[3] Kim T., Kim Y.-W., Cho H. (2020). Dynamic production scheduling model under due date uncertainty in precast concrete construction. J. Clean. Prod..

[4] Li X.-J., Zheng Y. (2020). Using LCA to research carbon footprint for precast concrete piles during the building construction stage: A China study. J. Clean. Prod..

[5] Türkel S., Alabas V. (2005). The effect of excessive steam curing on portland composite cement concrete. Cem. Concr. Res..

[6] Shen P., Lu L., He Y., Wang F., Hu S. (2019). The effect of curing regimes on the mechanical properties, nano-mechanical properties and microstructure of ultra-high performance concrete. Cem. Concr. Res..

[7] Nie S., Zhang W., Hu S., Liu Z., Wang F. (2018). Improving the fluid transport properties of heat-cured concrete by internal curing. Constr. Build. Mater..

[8] Mak S.L., Ritchie D.J., Shapiro G., Banks R.W. Rapid microwave curing of precast concrete slab elements. Proceedings of the 5th CANMET/ACI International Conference on Recent Advances in Concrete Technology.

[9] Fuhrmann J.M. (2002). Micro-Curing. Concrete Construction. https://www.concreteconstruction.net/how-to/concrete-production-precast/micro-curing_o.

[10] Sumra Y., Payam S., Zainah I., Huzaifa H., Panjehpour M. (2019). Crossover Effect in Cement-Based Materials: A Review. Appl. Sci..

[11] Yang K.-H., Mun J.-S., Cho M.-S. (2015). Effect of Curing Temperature Histories on the Compressive Strength Development of High-Strength Concrete. Adv. Mater. Sci. Eng..

[12] Neville A.M., Brooks J.J. (1990). Concrete Technology.

[13] Carino N.J., Malhotra V.M. (2003). Handbook on Nondestructive Testing of Concrete.

[14] Escalante-García J.I., Sharp J.H. (1998). Effect of temperature on the hydration of the main clinker phases in portland cements: Part i, neat cements. Cem. Concr. Res..

[15] Ogirigbo O.R., Black L. (2016). Influence of slag composition and temperature on the hydration and microstructure of slag blended cements. Constr. Build. Mater..

[16] Ezziane K., Bougara A., Kadri A., Khelafi H., Kadri E. (2007). Compressive strength of mortar containing natural pozzolan under various curing temperature. Cem. Concr. Compos..

[17] Kjellsen K., Detwiler R. (1993). Later-age strength prediction by a modified maturity model. ACI Mater. J..

[18] Newman J., Choo B.S. (2003). Advanced Concrete Technology 3: Processes.

[19] Khan M.S., Abbas H. (2016). Performance of concrete subjected to elevated temperature. Eur. J. Environ. Civ. Eng..

[20] Lothenbach B., Winnefeld F., Alder C., Wieland E., Lunk P. (2007). Effect of temperature on the pore solution, microstructure and hydration products of Portland cement pastes. Cem. Concr. Res..

[21] Sajedi F., Razak H.A. (2010). Thermal activation of ordinary Portland cement–slag mortars. Mater. Des..

[22] Kim J.-K., Han S.H., Song Y.C. (2002). Effect of temperature and aging on the mechanical properties of concrete: Part I. Experimental results. Cem. Concr. Res..

[23] Namarak C., Satching P., Tangchirapat W., Jaturapitakkul C. (2017). Improving the compressive strength of mortar from a binder of fly ash-calcium carbide residue. Constr. Build. Mater..

[24] Novotny M., Skramlik J., Suhajda K., Tichomirov V. (2013). Sterilization of Biotic Pests by Microwave Radiation. Procedia Eng..

[25] Procházka M., Sobotka J., Šuhajda K., Novotný M. (2016). Microwave radiation and its application on construction materials. Eng. Struct. Technol..

[26] (2004). Directive 2004/40/EC of the European Parliament and of the Council of 29 April 2004 on the Minimum Health and Safety Requirements Regarding the Exposure of Workers to the Risks Arising from Physical Agents (Electromagnetic Fields) (18th Individual Directive within the meaning of Article 16(1) of Directive 89/391/EEC). OJEU.

[27] Makul N. (2020). Effect of low-pressure microwave-accelerated curing on the drying shrinkage and water permeability of Portland cement pastes. Case Stud. Constr. Mater..

[28] Kvapilová V. (2020). Evaluation of microwave drying effects on historical brickwork and modern building materials. IOP Conf. Ser. Mater. Sci. Eng..

[29] Kvapilová V., Šuhajda K. (2020). Possibility of Using Microwave Radiation for Rehabilitation of Historical Masonry Constructions. Key Eng. Mater..

[30] Tauhiduzzaman M., Hafez I., Bousfield D., Tajvidi M. (2021). Modeling Microwave Heating and Drying of Lignocellulosic Foams through Coupled Electromagnetic and Heat Transfer Analysis. Processes.

[31] Průša D., Šuhajda K., Žajdlík T., Svobodová K., Šťastník S., Hobzova K., Venkrbec V. (2023). Effect of Microwave Radiation on the Compressive Strength of Solid Ceramic Brick. Buildings.

[32] Kääriäinen H., Rudolph M., Schaurich D., Tulla K., Wiggenhauser H. (2001). Moisture measurements in building materials with microwaves. NDT E Int..

[33] (2020). Testing Hardened Concrete—Part 7: Density of Hardened Concrete.

[34] (2020). Testing Hardened Concrete—Part 5: Flexural Strength of Test Specimens.

[35] (2020). Testing Hardened Concrete—Part 3: Compressive Strength of Test Specimens.

[36] (2021). Concrete—Specification, Performance, Production and Conformity.

